# Effects of fermentation on protein profile of coffee by-products and its relationship with internal protein structure measured by vibrational spectroscopy

**DOI:** 10.5713/ab.22.0469

**Published:** 2023-05-02

**Authors:** Samadi, Xin Feng, Luciana Prates, Siti Wajizah, Zulfahrizal, Agus Arip Munawar, Peiqiang Yu

**Affiliations:** 1 Animal Husbandry Department, Agricultural Faculty, Universitas Syiah Kuala, Darussalam - Banda Aceh, 23111, Indonesia; 2 Department of Animal and Poultry Science, College of Agriculture and Bioresources, University of Saskatchewan, 51 Campus Drive, Saskatoon, SK, S7N 5A8, Canada; 3 Department of Agricultural Engineering, Universitas Syiah Kuala, Darussalam -Banda Aceh 23111, Indonesia

**Keywords:** By-product, Coffee Processing, Fermentation, Nutrient Availability, Protein Molecular Structure, Vibrational Spectroscopy

## Abstract

**Objective:**

To our knowledge, there are few studies on the correlation between internal structure of fermented products and nutrient delivery from by-products from coffee processing in the ruminant system. The objective of this project was to use advanced mid-infrared vibrational spectroscopic technique (ATR-FT/IR) to reveal interactive correlation between protein internal structure and ruminant-relevant protein and energy metabolic profiles of by-products from coffee processing affected by added-microorganism fermentation duration.

**Methods:**

The by-products from coffee processing were fermented using commercial fermentation product, called Saus Burger Pakan, consisting of various microorganisms: cellulolytic, lactic acid, amylolytic, proteolytic, and xylanolytic microbes, for 0, 7, 14, 21, and 28 days. Protein chemical profiles, Cornell Net Carbohydrate and Protein System crude protein and CHO subfractions, and ruminal degradation and intestinal digestion of protein were evaluated. The attenuated total reflectance-Ft/IR (ATR-FTIR) spectroscopy was used to study protein structural features of spectra that were affected by added microorganism fermentation duration. The molecular spectral analyses were carried using OMNIC software. Molecular spectral analysis parameters in fermented and non-fermented by-products from coffee processing included: Amide I area (AIA), Amide II (AIIA) area, Amide I heigh (AIH), Amide II height (AIIH), α-helix height (αH), β-sheet height (βH), AIA to AIIA ratio, AIH to AIIH ratio, and αH to βH ratio. The relationship between protein structure spectral profiles of by-products from coffee processing and protein related metabolic features in ruminant were also investigated.

**Results:**

Fermentation decreased rumen degradable protein and increased rumen undegradable protein of by-products from coffee processing (p<0.05), indicating more protein entering from rumen to the small intestine for animal use. The fermentation duration significantly impacted (p<0.05) protein structure spectral features. Fermentation tended to increase (p<0.10) AIA and AIH as well as β-sheet height which all are significantly related to the protein level.

**Conclusion:**

Protein structure spectral profiles of by-product form coffee processing could be utilized as potential evaluators to estimate protein related chemical profile and protein metabolic characteristics in ruminant system.

## INTRODUCTION

Coffee (*Coffea Arabica* L.) is one of the most popular beverages in the world. The coffee fruit, also called cherry, from coffee tree is a reddish two-seeded berry which contains six layers: coffee bean, silverskin, parchment, mucilage, pulp, and skin. The coffee by-product, also identified as coffee fruit without beans, is an abundant agricultural by-product. Coffee waste and by-products cause serious environmental problem thus different methods have been developed to utilize the coffee by-products as raw material to produce feed, biogas, pectin, protein, and compost. The coffee by-products comprise nearly 45% of the cherry and can be used for food industry and agro-industry, such as extraction of caffeine and polyphenols [1]. Green coffee, covered or not with the silverskin, is the coffee internationally traded and is produced by either dry or wet processing [1]. The coffee by-products from dry processing are composed by the skin, pulp, mucilage and parchment in one single fraction [2]. However, in wet processing, usually the skin and pulp are in one fraction (43.2% w/w from the whole fruit), mucilage and soluble sugars in a second fraction (11.8% w/w) and the last fraction as parchment (6.1%) [3].

More than 50% of coffee by-products have been used for energy production, adsorption of compounds, manufacturing of industrial products such as particleboards [1]. Coffee pulp and husk are rich in carbohydrates (35% to 85%), soluble fibers (30.8%), minerals (3% to 11%), proteins (5% to 11%) and bioactive compounds such as caffeine and polyphenols [4]. Further exploration of these high value by-products as food additives or supplements is economically attractive. Most of the research have been focusing on using coffee by-products as healthy promoting food ingredients [5–8]. Coffee by-products can also be used for production of mushrooms, vermicomposting, ethanol, biogas, compost, dyes among others [9]. With feed cost increasing, interests on discovery of new and cheap feed for animals bring attention of scientists on agricultural by-products. The agricultural residues are abundant and inexpensive. Thus, it can avoid competition with human food and brings economic feasibility. Direct use of coffee by-products for animal feed is not possible due to the anti-physiological and anti-nutritional factors (e.g. tannins and caffeine) [10]. After fermentation, total phenolic compounds substantially decreased compared with the concentration in coffee pulp without undergoing fermentation [9,11]. With fermentation process detoxifying the coffee pulp, the fermented residues can be used as animal feed.

The chemical and nutritional properties of coffee by-products as feed for ruminants are less studied. Biochemical analysis using newly developed techniques as well as nutritional prediction models will help identifying its feeding values on animals. The relationship between nutrient inherent molecular structure and the nutritive values have been reported in many published studies. The intrinsic molecular structure of nutrients such as protein and carbohydrates has been proposed to be potential predictors to estimate nutrient supply and availability for ruminants [12,13]. The attenuated total reflectance-Fourier transform infrared (ATR-FTIR) spectroscopy is a rapid, non-destructive analytical approach. It has been used to study nutrient molecular structure, nutrient utilization, and nutrient availability [12]. Minimal sample preparation and no chemical necessary during analysis are among its advantages. The objectives of current study were: i) to evaluate the effects of fermentation on protein chemical profiles, Cornell Net Carbohydrate and Protein System (CNCPS) subfractions, ruminal and intestinal digestion of coffee by-products; ii) to study the effects of fermentation on protein molecular structure of coffee-byproducts using advanced molecular ATR-FTIR spectroscopy; and iii) to associate fermentation induced changes in protein metabolic characteristics with protein molecular structure changes.

## MATERIALS AND METHODS

The University of Saskatchewan Animal Care Committee approved the animal trial under the Animal Use Protocol No. 19910012 and animals were cared for and handled in accordance with the Canadian Council of Animal Care (CCAC, 1993) regulations. Authors confirm that EU and Canadian standards for the protection of animals and/or feed legislation have been met.

### Microorganism fermentation study

Coffee by-products (skin and pulp) were collected from coffee plantations (wet processing). Collected by-products were dried to reach 90% of dry matter (DM) in 60°C oven for 24 h. The commercial fermentation product, called Saus Burger Pakan (SBP; 1%), was used for fermentation. The SBP product consists of various microorganisms (cellulolytic, lactic acid, amylolytic, proteolytic, and xylanolytic microbes) and some herbal extracts. Before SBP was mixed with coffee by-products, it was mixed with 3% molasses for 5 h to activate the microbes. The samples were ground and fermented with SBP for 0, 7, 14, 21, and 28 d. Sago was added as energy source to maximize microbial fermentation (3%). To reach water content 40% for the fermentation mix, water was also added. The fermentation study was conducted using air-tight containers under room temperature (28°C to 30°C). Total of 15 containers were prepared with three containers for each time point.

At each fermentation time point, three containers were opened, and contents were mixed thoroughly before being dried in air-ventilated oven (60°C) until constant weight was reached. Dried fermented and non-fermented samples were ground with a Retsch mill (Retsch ZM-200; Brinkmann Instruments Ltd., Mississauga, Canada) for chemical composion analysis (1 mm screen) and structure spectral analysis (0.12 mm screen).

### Chemical analysis of the fermented and non-fermented by-products

The fermented and non-fermented by-product samples were analyzed using AOAC method [14] for DM (930.15), crude protein (CP 984.13), ash (942.05), ether extract (EE 920.39). Soluble crude protein (SCP) was analyzed using the method published by Roe et al [15]. The neutral detergent insoluble protein (NDICP) and acid detergent insoluble protein (ADICP) were analyzed using the published method [16].

### Protein subfractions, ruminal and intestinal protein digestion estimation with updated CNCPS system

The protein subfractions, protein ruminal degradable subfractions and undegradable subfractions of the fermented and non-fermented by-product samples were evaluated with the updated Cornell Net Carbohydrate and Protein System-CNCPS 6.5 [17]. The subfractions include: rapidly degradable PA2 fraction, intermediately degradable PB1 fraction, slowly degradable PB2 and indigestible PC fraction. The PC was the undegradable part which was equal to ADICP and it was often combined with lignin and tannin. The rates of ruminal degradation (Kd) are ca. 10%/h to 40%/h for PA2, 3%/h to 20%/h for PB1, and 1%/h to 18%/h for PB2 [18].

### Protein molecular spectra collection and analysis using vibrational molecular spectroscopy for the fermented and non-fermented by-products

The protein internal spectral parameters were determined with a JASCO FT/IR-ATR-4200 spectroscopy (JASCO Corp., Tokyo, Japan) at the SRP Chair molecular spectroscopy lab (University of Saskatchewan, Saskatoon, Canada). Spectra of the fermented and non-fermented by-product samples were obtained in the mid-infrared range from ca. 4,000 to 700 cm^−1^ with total 256 scans per spectrum at the resolution of 4 cm^−1^. The unique protein structure spectral peak bands were identified [19] including: amide I (region ca. 1,708 to 1,588 cm^−1^; peak ca. 1,610 cm^−1^), amide II (region ca. 1,588 to 1,488 cm^−1^; peak ca. 1,536 cm^−1^), α-helix (ca. 1,650 cm^−1^) and β-sheet (ca. 1,637 cm^−1^). The α-helix and β-sheet were identified by using both second derivative and FSD spectral functions. The OMNIC 7.3 spectral collection and analysis software (Thermo Electron Corp., Madison, WI, USA) was applied for quantification of absorption peaks area and peak heights of spectral bands that were related to protein primary and secondary structures.

### Statistical analysis

In this project, the data from above studies were analyzed using the MIXED model procedure of SAS (SAS Institute, Inc., Cary, NC, USA) with fermentation duration as fixed effects. The residual analysis was carried out to check the model assumptions. Normality check was conducted using SAS Univariate procedure with Normal and Plot options. Multiple comparisons were carried out using Tukey method. Preplanned contrasts were used to evaluate linear, quadratic, and cubic treatment effects of treatment. The contrast of no fermentation (d0) and fermentation (average of fermentation for 7, 14, 21, and 28 days) was used to evaluate the overall difference between the fermentation and without fermentation. Significant differences were declared at p<0.05 and trends at p<0.10.

Correlation of the spectral features of protein structure in by-products with protein metabolic characteristics were performed using the CORR procedure of SAS software. The Spearman option was used because some of the data was not normally distributed which detected using Univariate procedure with Normal and Plot options in SAS. The stepwise multiple regression analyses were conducted using the PROC REG procedure. Protein molecular spectral features were used to estimate protein related characteristics. Only independent variable parameters contributing significantly (p< 0.05) to the dependent variable were retained in the final model.

## RESULTS AND DISCUSSION

### Fermentation effects on protein chemical profiles of by-products from coffee processing

The chemical composition profiles including protein profile data of coffee by-products after different fermentation duration are showed in Table 1. Dry matter content of fermentation products from different time points were different. The DM content after fermentation for 14, 21, and 28 days decreased compared to the group without fermentation. The EE content linearly increased as fermentation time increased (p = 0.002; Table 1). The relatively higher protein in by-products that were added fermentation product in comparison with the control was mainly due to added microorganism that contained protein. This might be due to microbes activity [20]. The SCP content was decreased (p<0.05) linearly with fermentation duration. The possible reason could be that the microbes incorporate soluble protein into its own cell structure thus decreased the SCP in the environment. Fermentation with SBP did not affect NDICP neither expresses as percent of DM nor CP. The ADICP (% DM or % CP) linearly increased with fermentation duration. Fermentation has become one of the most important approaches to treat waste and agricultural by-products to minimize the environmental pollution as well as to reduce cost of production. Various researchers have reported using fermentation to produce pectinase [21], fructosyl transferase [22], polyhydroxyalkanoates [20], and biosurfactant [23]. Limited fermentation studies have been conducted on coffee by-products (skin and pulp) from protein characteristics’ perspective. The current study can provide some preliminary data on fermented coffee by-products as feed for ruminants.

### Fermentation effects on protein subfractions, ruminal and intestinal protein digestion of by-products from coffee processing

The CNCPS system was developed to predict nutrient requirements, feed utilization, animal performance and nutrient excretion for dairy and beef cattle using accumulated knowledge about feed composition, feed degradation, feed digestion, and feed metabolism. The CNCPS feed library consists of approximately 800 ingredients, including forages, concentrations, vitamins, minerals, and commercial products [24]. It is the next best method to measure nutrients digestion and utilization in ruminants after animal experiments with its relatively accurate prediction ability. In the current study, the CNCPS was used to partition protein subfractions of fermentation products and predict ruminal and intestinal protein digestion. Many studies have been using CNCPS to partition protein of various kinds of seeds [12,18,19]. To our best knowledge, this was the first time to use CNCPS to partition protein of fermented coffee by-products.

The soluble true protein fraction, PA2, linearly decreased as the same as SCP since PA2 was calculated as PA2 = SCP. The insoluble true protein PB1 linearly increased with fermentation. The microbes utilize soluble protein to make microbial protein which was not soluble, this could be the reason that PB1 increased linearly. The PB2 fraction was not affected by fermentation and PC fraction was the same as ADICP. The protein rumen degradation was presented in Table 2. The groups fermented for 7 and 14 days had the highest CP content than other groups. As fermentation time increased, total rumen undegradable protein linearly increased (p = 0.065; p<0.001). This is favorable in ruminants as more RUP entering the small intestine can maximize the feed protein utilization by the animals [24]. Rumen degradable soluble protein (RDPA2) was linearly decreased and rumen degradable insoluble true protein (RDPB1) linearly increased with fermentation duration increased. The rumen degradable fiber-bound protein (RUPB2) was not affected by fermentation (p = 0.155). Predicted rumen undegradable soluble true protein (RUPA2) and rumen undegradable insoluble true protein (RUPB1) linearly decreased and increased, respectively (Table 4). Rumen undegradable indigestible protein (RUPC), the portion not digestible, was the same as PC fraction and it linearly increased with fermentation. Digestion data of the rumen bypass protein in the post-ruminal intestinal tract was presented in Table 2. Intestinal digestion of soluble true protein (IDPA2) linearly decreased and intestinal digestion of insoluble true protein (IDPB1) linearly increased with fermentation time increased. One of the purposes of feed processing was to slow down the protein degradation by ruminal microbes and increase the rumen bypass protein available for animal use [25]. Intestinal digestion of feed protein (IDFP) linearly increased as fermentation time increased indicating that fermentation with SBP increased protein available for the animals.

### Fermentation effects on protein molecular spectral profiles of by-products from coffee processing

The advanced molecular spectroscopy methods have been developed in recent years and successfully used to quantitatively evaluate the primary and secondary molecular structures of feed protein [13,25]. The structure spectral features of amide in this study mainly include the amides I and II areas and their heights, secondary structures of α-helix and β-sheet. Fermentation tended to affect amide I peak area and peak height as well as β-sheet peak height. The amide II peak area and peak height as well as α-helix peak height were not different among five groups. None of the ratios of protein molecular structural spectral intensity were affected by fermentation (Table 3). Numerous research studies have illustrated that protein amide profile and secondary structure profiles are highly associated with biological value and quality of protein to ruminants [25] as the molecular structure of protein affect ruminal and intestinal CP digestion. It was reported that the molecular structure of protein is highly related to the protein solubility and its access to microbes and digestive enzymes [13]. Usually the amide profiles in terms of peak area and height reveal information about the concentration of protein in feed [13].

### Interactive association: correlation and regression studies of protein spectral features and metabolic profiles of by-products from coffee processing

Table 4 presents the spearman correlation coefficients between protein molecular spectral features and protein related profiles. Table 5 presents the multiple regression equations using protein spectral characteristics and their predictive power for the protein related metabolic profiles. Amide I peak area and peak height had significantly negative correlation with SCP, PA2, RDPA2, RUPA2, and IDPA2. The correlation analysis results suggests that protein internal structure features could highly influence protein degradation in the rumen and post-ruminal digestive tract [12]. These are consistent with the regression equations presented in Table 5. The independent variable amide I height (AIH) was the only predictor left for predicting these dependent variables. And increasing AIH decreased the values of these variables (Table 6). Amide I spectral intensity peak area and peak height had significantly positive correlation with ADICP, PB1, PC, RUPB1, and IDPB1 for which amide I intensity peak area (AIA) was left in the final evaluation models. The spectral intensity height of α-helix and β-sheet had significantly negative correlations with SCP, PA2, RDPA2, RUPA2, IDPA2 and significantly positive correlation with PB1, RDPB1, RUPB1, and IDPB1. Other spectral features (amide II peak area and height, peak intensity area ratio of amide I to II, peak intensity height ratio of amide I to II, peak intensity height ratio of α-helix to β-sheet) did not show any correlations with any of the protein related profiles. The peak area and peak height of amide I were the only two spectral features retained in the final evaluation model. All the regression models have relatively lower R squares. Khan et al [26] reported that the amide I to amide II ratio and α-helix to β-sheet ratio were better predictors of protein nutritive value and digestion. However, this was not observed in the current study. The inconsistency with current study might be cause by different feeds. To our knowledge, this is the first study of relationship between the internal structure spectral features and metabolic characteristics of protein in by-products from coffee-processing impacted by added-microorganism fermentation duration. We would not have available data to compare. It is noteworthy to mention that these prediction equations are based on limited samples. To establish the prediction equation for future application, a large number of samples with diverse source of coffee by-products would be required.

## CONCLUSION

Fermentation of coffee by-products with the SBP additive increased the RUP entering post ruminal digestive tract as well as intestinal protein digestion. Fermentation also tended to increase spectral amide I intensity peak area and peak height and β-sheet spectral peak height which are related to the protein functional group and structure. Internal structure spectral features of protein in by-products from coffee processing could be used as potential evaluators for estimation of protein related nutritional characteristics in ruminant system.

## Figures and Tables

**Table 1:** Effect of fermentation duration on protein chemical profiles of coffee by-products

Item	Fermentation duration (d)					SEM	p-value	Contrast^1)^			
	
	F0	F7	F14	F21	F28			F0 vs F	L	Q	C
Basic chemical (% DM)											
DM	63.66^a^	61.80^ac^	59.60^bc^	60.56^bc^	59.29^bc^	0.895	0.021	0.004	0.175	0.005	0.660
NDF	37.49^b^	39.56^a^	37.52^b^	37.60^ab^	38.90^ab^	0.436	0.019	0.092	0.539	0.728	0.003
ADF	28.88^b^	30.96^a^	28.41^b^	29.10^ab^	29.15^ab^	0.438	0.018	0.307	0.361	0.620	0.160
EE	1.93^ab^	1.71^b^	1.99^ab^	2.04^ab^	2.22^a^	0.070	0.006	0.433	0.002	0.060	0.144
Protein profile (% DM)											
CP	14.00	14.83	14.56	14.04	14.63	0.197	0.050	0.040	0.457	0.340	0.005
SCP	7.03^b^	8.11^a^	6.69^b^	6.35^b^	6.59^b^	0.212	0.001	0.705	0.002	0.465	0.001
SCP (% CP)	50.19^b^	54.71^a^	45.95^c^	45.17^c^	45.06^c^	0.88	<0.001	0.031	<0.001	0.710	<0.001
NDICP	3.60	3.61	2.95	3.14	3.54	0.169	0.058	0.151	0.285	0.027	0.128
NDICP (% CP)	20.58	18.04	18.14	20.27	21.79	0.941	0.071	0.357	0.147	0.016	0.300
ADICP	1.30^b^	1.32^b^	1.54^a^	1.51^a^	1.58^a^	0.031	<0.001	<0.001	<0.001	0.305	0.344
ADICP (%CP)	9.34^bc^	8.92^c^	10.60^ab^	10.74^a^	10.85^a^	0.275	0.001	0.012	<0.001	0.646	0.033

SEM, standard error of the mean; DM, dry matter; NDF, neutral detergent fibre; ADF, acid detergent fibre; EE, ether extract; CP, crude protein; SCP, soluble crude protein; NDICP, neutral detergent insoluble CP; ADICP, acid detergent insoluble CP.

1) Polynomial contrast: L, linear; Q, quadratic; C, cubic.

a–c Means with different letters within the same row differ (p<0.05).

**Table 2:** Effect of fermentation duration on protein subfractions, rumen degradable and undegradable protein subfractions, and estimated intestinal digestion of protein of coffee by-products (CNCPS 6.5)

Item^1)^	Fermentation duration (d)					SEM	p-value	Contrast^2)^			
	
	F0	F7	F14	F21	F28			F0 vs F	L	Q	C
CNCPS protein subfractions (% DM)											
PA2	7.03^b^	8.11^a^	6.69^b^	6.35^b^	6.59^b^	0.212	0.001	0.705	0.002	0.465	0.001
PB1	4.08^b^	4.04^b^	5.22^a^	4.84^a^	4.84^a^	0.148	<0.001	0.002	<0.001	0.024	0.099
PB2	1.57	1.35	1.10	1.33	1.60	0.140	0.155	0.182	0.919	0.019	0.897
PC	1.30^b^	1.32^b^	1.54^a^	1.51^a^	1.58^a^	0.031	<0.001	<0.001	<0.001	0.305	0.344
Rumen degradable protein (% DM)											
RDPA2	5.02^b^	5.79^a^	4.78^b^	4.53^b^	4.71^b^	0.151	0.001	0.705	0.002	0.465	0.001
RDPB1	1.63^b^	1.61^b^	2.09^a^	1.93^a^	1.93^a^	0.059	<0.001	0.002	<0.001	0.024	0.099
RDPB2	0.52	0.45	0.36	0.44	0.53	0.046	0.155	0.182	0.920	0.019	0.897
Total RDP	7.18^b^	7.86^a^	7.24^ab^	6.92^b^	7.18^b^	0.143	0.010	0.470	0.065	0.345	0.001
Rumen undegradable protein (% DM)											
RUPA2	2.00^b^	2.31^a^	1.91^b^	1.81^b^	1.88^b^	0.060	0.001	0.704	0.002	0.466	0.001
RUPB1	2.45^b^	2.42^b^	3.13^a^	2.90^a^	2.90^a^	0.089	<0.001	0.002	<0.001	0.024	0.099
RUPB2	1.04	0.90	0.73	0.89	1.06	0.093	0.155	0.182	0.919	0.019	0.897
RUPC	1.30^b^	1.32^b^	1.54^a^	1.51^a^	1.58^a^	0.031	<0.001	<0.001	<0.001	0.305	0.344
Total RUP	6.81^d^	6.96^cd^	7.32^ab^	7.12^bc^	7.45^a^	0.060	<0.001	<0.001	<0.001	0.382	0.134
Intestinal digestibility (% DM)											
IDPA2	2.00^b^	2.31^a^	1.91^b^	1.81^b^	1.88^b^	0.060	0.001	0.704	0.002	0.466	0.001
IDPB1	2.45^b^	2.42^b^	3.13^a^	2.90^a^	2.90^a^	0.089	<0.001	0.002	<0.001	0.024	0.099
IDPB2	0.83	0.72	0.58	0.71	0.85	0.075	0.155	0.182	0.920	0.019	0.897
IDFP	5.30^b^	5.46^ab^	5.63^a^	5.43^ab^	5.64^a^	0.067	0.021	0.008	0.010	0.298	0.086

CNCPS, Cornell Net Carbohydrate and Protein System; SEM, standard error of the mean; DM, dry matter.

1) PA2, soluble true protein; PB1, insoluble true protein; PB2, fiber-bound protein; PC, indigestible protein; RD: predicted rumen degradable soluble true protein (RDPA2); insoluble true protein (RDPB1); fiber-bound protein (RDPB2); RU: predicted rumen undegradable soluble true protein (RDPA2); insoluble true protein (RUPB1); fiber-bound protein (RUPB2); indigestible protein (RUPC); RDP, predicted rumen degradable protein; RUP, predicted rumen undegradable protein; ID: intestinal digestion of the protein subfractions PA2: soluble true protein (IDPA2); PB1: insoluble true protein (IDPB1); PB2: fibre-bound protein (IDPB2) partitioned and feed protein (IDFP).

2) Polynomial contrast: L, linear; Q, quadratic; C, cubic.

a–d Means with different letters within the same row differ (p<0.05).

**Table 3:** Effect of fermentation duration on protein molecular spectral features of coffee by-products

Item	Fermentation duration (d)					SEM	p-value	Contrast^1)^			
	
	F0	F7	F14	F21	F28			C vs F	L	Q	C
Protein amide sructure spectral profile											
Amide I area	19.52	20.17	21.07	22.04	21.69	0.625	0.09	0.76	0.03	0.09	0.30
Amide II area	2.33	2.84	2.74	2.66	2.63	0.185	0.43	0.55	0.30	0.40	0.22
Amide I height	0.225	0.230	0.241	0.25	0.245	0.006	0.093	0.66	0.03	0.144	0.257
Amide II height	0.079	0.095	0.094	0.070	0.082	0.010	0.418	0.291	0.856	0.665	0.071
Protein secondary structure											
α-Helix height	0.152	0.158	0.166	0.175	0.169	0.006	0.162	0.669	0.044	0.180	0.379
β-Sheet height	0.193	0.202	0.208	0.223	0.213	0.006	0.080	0.99	0.040	0.083	0.222
Ratios of protein molecular structural spectral intensity											
Amide I:II area	8.46	7.12	7.85	8.29	8.36	0.592	0.510	0.765	0.983	0.824	0.145
Amide I:II height	3.02	2.46	2.66	3.54	3.01	0.319	0.238	0.355	0.726	0.587	0.033
α-Helix:β-sheet	0.786	0.785	0.801	0.783	0.791	0.009	0.635	0.170	0.349	0.481	0.519

SEM, standard error of the mean.

1) Polynomial contrast: L, linear; Q, quadratic; C, cubic.

**Table 4:** Correlation analyses between protein structure spectral features and protein metabolic profiles of fermented coffee by-products using fourier transform/infrared-attenuated total reflectance molecular spectroscopy (ATR-FTIR)

Variable	AIA	AIIA	AIH	AIIH	αH	βH	AAratio	AHratio	αβratio
Protein chemical profile (% DM)									
CP	−0.057	0.20	−0.032	0.25	−0.05	−0.042	−0.25	−0.31	0.075
SCP	−0.69^*^	0.033	−0.66^*^	0.19	−0.67^*^	−0.69^*^	−0.31	−0.39	−0.27
SCP (% CP)	−0.69^*^	−0.030	−0.64^*^	0.12	−0.67^*^	−0.67^*^	−0.24	−0.33	−0.36
NDICP	−0.48	−0.030	−0.51	0.11	−0.48	−0.50	−0.069	−0.24	−0.19
NDICP (% CP)	0.014	−0.18	−0.054	−0.18	−0.046	−0.036	0.24	0.18	−0.092
ADICP	0.22	−0.071	0.20	0.039	0.17	0.16	−0.017	−0.086	−0.007
ADICP (% CP)	0.56^*^	−0.14	0.51^*^	−0.096	0.48	0.50	0.24	0.19	0.054
CNCPS subfractions (% DM)									
PA2	−0.60^*^	0.037	−0.57^*^	0.13	−0.57^*^	−0.60^*^	−0.28	−0.33	−0.12
PB1	0.53^*^	0.20	0.52^*^	0.15	0.53^*^	0.52^*^	−0.028	0.021	0.39
PB2	−0.32	−0.032	−0.37	0.057	−0.33	−0.33	0.039	−0.12	−0.11
PC	0.55^*^	−0.22	0.48	−0.11	0.46	0.45	0.31	0.21	0.23
Rumen degradable protein (% DM)									
RDPA2	−0.60^*^	0.038	−0.57^*^	0.13	−0.57^*^	−0.61^*^	−0.28	−0.33	−0.12
RDPB1	0.53^*^	0.20	0.52^*^	0.15	0.53^*^	0.52^*^	−0.029	0.021	0.39
RDPB2	−0.32	−0.032	−0.37	0.057	−0.33	−0.33	0.039	−0.12	−0.11
Total RDP	−0.48	0.13	−0.46	0.24	−0.45	−0.50	−0.28	−0.38	0.03
Rumen undegradable protein (% DM)									
RUPA2	−0.60^*^	0.038	−0.57^*^	0.13	−0.57^*^	−0.61^*^	−0.28	−0.33	−0.12
RUPB1	0.53^*^	0.20	0.52^*^	0.15	0.53^*^	0.52^*^	−0.029	0.021	0.39
RUPB2	−0.32	−0.032	−0.37	0.057	−0.33	−0.33	0.039	−0.12	−0.11
RUPC	0.55^*^	−0.22	0.48	−0.11	0.46	0.45	0.31	0.21	0.23
Total RUP	0.40	0.071	0.37	0.082	0.40	0.39	0.082	0.011	0.37
Intestinal digestibility (% DM)									
IDPA2	−0.60^*^	0.038	−0.57^*^	0.13	−0.57^*^	−0.61^*^	−0.28	−0.33	−0.12
IDPB1	0.53^*^	0.20	0.52^*^	0.15	0.53^*^	0.52^*^	−0.029	0.021	0.39
IDPB2	−0.33	−0.032	−0.37	0.057	−0.33	−0.33	0.039	−0.12	−0.11
IDFP	0.23	0.20	0.26	0.25	0.31	0.29	−0.088	−0.18	0.37

AIA, amide I area; AIIA, amide II area; AIH, amide I height; AIIH, amide II height; Ah, α-helix height; βH, β-sheet height; AAratio, ratio of amide I area to amide II area; AHratio, ratio of amide I height to amide II height; αβratio, ratio of α-helix height to β-sheet height; CP, crude protein; SCP, soluble crude protein; NDICP, neutral detergent insoluble CP; ADICP, acid detergent insoluble CP; PA2, soluble true protein; PB1, insoluble true protein; PB2, fiber-bound protein; PC, indigestible protein; RD: predicted rumen degradable soluble true protein (RDPA2); insoluble true protein (RDPB1); fiber-bound protein (RDPB2); RU: predicted rumen undegradable soluble true protein (RDPA2); insoluble true protein (RUPB1); fiber-bound protein (RUPB2); indigestible protein (RUPC); RDP, predicted rumen degradable protein; RUP, predicted rumen undegradable protein; ID: intestinal digestion of the protein subfractions PA2: soluble true protein (IDPA2); PB1: insoluble true protein (IDPB1); PB2: fibre-bound protein (IDPB2) partitioned and feed protein (IDFP).

* p<0.05.

**Table 5:** Multiple regression equations for evaluating protein metabolic profiles of fermented by-products from coffee processing using protein molecular structure spectral features

Variables	Equations	Model R^2^ value	RMSE	p-value
Protein profile (% DM)				
CP	NA			
SCP	25.74–73.04×AIH	0.35	1.34	0.02
SCP (% CP)	96.59–202.8×AIH	0.41	3.25	0.009
NDICP	NA			
NDICP (% CP)	NA			
ADICP	NA			
ADICP (% CP)	1.03+0.43×AIA	0.39	0.74	0.012
Protein subfraction (% DM)				
PA2	14.47–31.48×AIH	0.33	0.60	0.025
PB1	−0.66+0.25×AIA	0.40	0.42	0.011
PB2	NA			
PC	0.22+0.06×AIA	0.37	0.11	0.016
Rumen degradable protein (% DM)				
RDPA2	10.33–22.49×AIH	0.33	0.43	0.025
RDPB1	−0.26+0.10×AIA	0.40	0.17	0.011
RDPB2	NA			
Total RDP	NA			
Rumen undegradable protein (% DM)				
RUPA2	4.13–8.99×AIH	0.33	0.17	0.025
RUPB1	−0.39+0.15×AIA	0.40	0.25	0.011
RUPB2	NA			
RUPC	0.22+0.059×AIA	0.37	0.11	0.016
Total RUP	NA			
Intestinal digestibility (% DM)				
IDPA2	4.13–8.99×AIH	0.33	0.17	0.025
IDPB1	−0.39+0.15×AIA	0.40	0.25	0.011
IDPB2	NA			
IDFP	NA			

RMSE, root mean square error; CP, crude protein; SCP, soluble crude protein; NDICP, neutral detergent insoluble CP; ADICP, acid detergent insoluble CP; PA2, soluble true protein; PB1, insoluble true protein; PB2, fiber-bound protein; PC, indigestible protein; RD: predicted rumen degradable soluble true protein (RDPA2); insoluble true protein (RDPB1); fiber-bound protein (RDPB2); RU: predicted rumen undegradable soluble true protein (RDPA2); insoluble true protein (RUPB1); fiber-bound protein (RUPB2); indigestible protein (RUPC); RDP, predicted rumen degradable protein; RUP, predicted rumen undegradable protein; ID: intestinal digestion of the protein subfractions PA2: soluble true protein (IDPA2); PB1: insoluble true protein (IDPB1); PB2: fibre-bound protein (IDPB2) partitioned and feed protein (IDFP); AIA, amide I area; AIIA, amide II area; AIH, amide I height; AIIH, amide II height; Ah, α-helix height; βh, β-sheet height; AAratio, ratio of amide I area to amide II area; AHratio, ratio of amide I height to amide II height; αβratio, ratio of α-helix height to β-sheet height.
